# Evaluating the Usability and Usefulness of a Mobile App for Atrial Fibrillation Using Qualitative Methods: Exploratory Pilot Study

**DOI:** 10.2196/humanfactors.8004

**Published:** 2018-03-15

**Authors:** Jaclyn Hirschey, Sunetra Bane, Moussa Mansour, Jodi Sperber, Stephen Agboola, Joseph Kvedar, Kamal Jethwani

**Affiliations:** ^1^ Partners Connected Health Partners Healthcare Boston, MA United States; ^2^ Heart Center Massachusetts General Hospital Boston, MA United States; ^3^ Harvard Medical School Cambridge, MA United States; ^4^ Massachusetts General Hospital Boston, MA United States

**Keywords:** nonvalvular atrial fibrillation, medication adherence, patient self-care, mobile application, exploratory research, pilot study, usability study, acceptability study, qualitative methods

## Abstract

**Background:**

Atrial fibrillation (AFib) is the most common form of heart arrhythmia and a potent risk factor for stroke. Nonvitamin K antagonist oral anticoagulants (NOACs) are routinely prescribed to manage AFib stroke risk; however, nonadherence to treatment is a concern. Additional tools that support self-care and medication adherence may benefit patients with AFib.

**Objective:**

The aim of this study was to evaluate the perceived usability and usefulness of a mobile app designed to support self-care and treatment adherence for AFib patients who are prescribed NOACs.

**Methods:**

A mobile app to support AFib patients was previously developed based on early stage interview and usability test data from clinicians and patients. An exploratory pilot study consisting of naturalistic app use, surveys, and semistructured interviews was then conducted to examine patients’ perceptions and everyday use of the app.

**Results:**

A total of 12 individuals with an existing diagnosis of nonvalvular AFib completed the 4-week study. The average age of participants was 59 years. All participants somewhat or strongly agreed that the app was easy to use, and 92% (11/12) reported being satisfied or very satisfied with the app. Participant feedback identified changes that may improve app usability and usefulness for patients with AFib. Areas of usability improvement were organized by three themes: app navigation, clarity of app instructions and design intent, and software bugs. Perceptions of app usefulness were grouped by three key variables: core needs of the patient segment, patient workflow while managing AFib, and the app’s ability to support the patient’s evolving needs.

**Conclusions:**

The results of this study suggest that mobile tools that target self-care and treatment adherence may be helpful to AFib patients, particularly those who are newly diagnosed. Additionally, participant feedback provided insight into the varied needs and health experiences of AFib patients, which may improve the design and targeting of the intervention. Pilot studies that qualitatively examine patient perceptions of usability and usefulness are a valuable and often underutilized method for assessing the real-world acceptability of an intervention. Additional research evaluating the AFib Connect mobile app over a longer period, and including a larger, more diverse sample of AFib patients, will be helpful for understanding whether the app is perceived more broadly to be useful and effective in supporting patient self-care and medication adherence.

## Introduction

Atrial fibrillation (AFib) is the most common type of heart arrhythmia [1]. It is estimated that in the United States between 2.7 to 6.1 million people currently have AFib and that 1 in 4 adults 40 years and older will develop AFib during their lifetime [1,2]. It is characterized by palpitations, dizziness, weakness, and dyspnea and associated with increased health care costs and mortality and reduced quality of life [3,4]. Additionally, individuals with AFib have a 4- to 5-fold increased risk of stroke [5].

To manage AFib stroke risk, more than half of all individuals in the United States with AFib are prescribed a nonvitamin K antagonist oral anticoagulant (NOAC) [6]. NOACs have several advantages over older vitamin K antagonist anticoagulant medications, such as warfarin, because of their lower risk for food and drug interactions, simpler dosing regimens, and lack of requirement for continuous blood monitoring [7]. However, medication nonadherence—a common issue among many chronic conditions—continues to be a challenge for NOAC treatment [8,9]. More than half of individuals on an NOAC for AFib do not meet the Pharmacy Quality Alliance adherence threshold of 80%, putting them at an increased risk for thrombus formation [9,10]. Additionally, while underanticoagulation may pose a greater risk for stroke, overanticoagulation can increase the risk of bleeding [11]. Thus, careful adherence to clinician-prescribed treatment is essential to keep within a therapeutic dosing range and prevent adverse events.

The need for strict treatment adherence, coupled with distressing symptoms and disease complexity, make patient self-care difficult [12]. Although the introduction of NOACs has reduced the patient burden associated with warfarin treatment, it has also highlighted the need for new tools that support self-care and treatment adherence in the absence of frequent clinical oversight [13,14]. Existing tools to support AFib anticoagulant treatment have largely focused on providing decision support to clinicians at the point of prescription [15-17]. Additional patient-facing tools that target medication adherence and long-term self-care may be valuable, particularly for patients taking NOACs [13].

**Figure 1:**
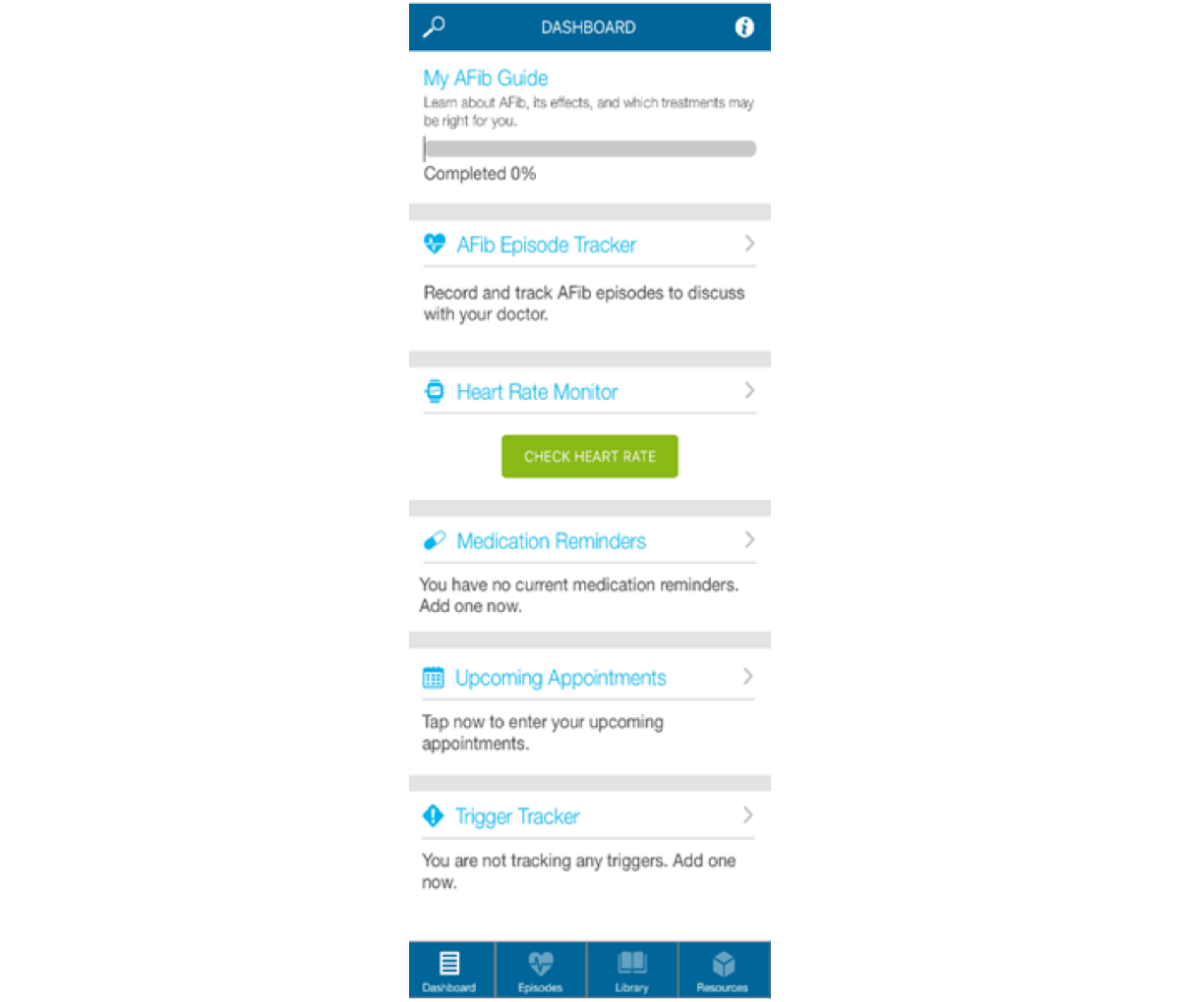
Dashboard screen of the AFib Connect app.

The AFib Connect mobile app (Figure 1), created for both Android and iPhone operating system (iOS) platforms, was developed with the goal of supporting long-term patient self-care and adherence to anticoagulant therapy. The app was developed by an interdisciplinary design team of clinicians, qualitative researchers, and user experience designers at Partners Connected Health in collaboration with Daiichi Sankyo, Inc. As part of a user-centered design approach, input from clinicians and patients was compiled to understand the primary goals, needs, and preferences for the app [18]. Semistructured interviews were conducted with nine AFib clinicians and patients to identify the app’s core features. An iterative process of feedback from key stakeholders was used to refine the app’s overall design. Usability testing was then conducted with clinicians and patients in a lab using the first version of the app. This feedback was incorporated back into the design of version two, which was used for this study. Table 1 outlines the features included in version two of the AFib Connect mobile app.

The goal of this pilot study was to evaluate the perceived usability and usefulness of the AFib Connect mobile app after an extended period of natural use by AFib patients prescribed an NOAC.

**Table 1:** AFib Connect app feature list and descriptions.

Features	Description
AFib^a^ Guide	An introduction to AFib through text and animated videos, including an overview of the condition, associated stroke risk, and a decision tool to review treatment options; information provided in the guide is based on the American Heart Association and Massachusetts General Hospital guidelines
Library	Detailed information on AFib, including types of medication available, procedure options, and guidance on medication adherence and stroke risk; information provided in the Library is based on the American Heart Association and Massachusetts General Hospital guidelines
Episode Tracker	Patient-generated log for tracking AFib episodes and associated notes for documentation and review with physician
Trigger Tracker	Patient-generated log for tracking possible episode triggers (eg, caffeine, alcohol, and poor sleep)
News Feed	Curated news content from five heart health–related Twitter feeds, such as American Heart News, StopAF.org, and the American Heart Association
Medication Reminder	Reminders to take medication at a designated time, including pop-up notifications, an option to mark medications as taken, and adherence history
Heart Rate Monitor	Tool for measuring heart rate using the mobile phone camera
Appointment Reminder	Calendar for tracking medical appointments and reminders of upcoming visits

^a^AFib: atrial fibrillation.

## Methods

This pilot study used qualitative methods and an exploratory research approach that combined naturalistic app use, surveys, and semistructured interviews to understand patient perceptions of the mobile app. A 4-week, five-visit study design gave participants the opportunity to use the app in their everyday environment and provide detailed feedback on select app features each week. The study received approval from the Partners HealthCare Human Research Committees and the institutional review board of Massachusetts General Hospital. All participants provided written consent and were compensated US $200 for their participation.

### Study Population

The study was conducted from September 2016 to April 2017. A purposeful sample of individuals diagnosed with nonvalvular AFib and taking NOACs to manage stroke risk were selected to participate. Potential participants were identified by clinician referral from the department of cardiology at Massachusetts General Hospital and contacted for recruitment. A total of 16 participants enrolled and 12 participants completed the 4-week study. Among the 4 participants who did not continue through study closeout, 2 were found ineligible after enrollment because of mobile phone operating system incompatibility, 1 was lost to follow-up after week 1, and 1 dropped out after enrollment because of a lack of interest. Enrollment of participants continued until thematic data saturation was reached.

### Data Collection

Surveys were administered in-person at study enrollment and by mail at closeout. Semistructured interviews were conducted in-person during the enrollment visit to establish rapport between the participant and the interviewers and by phone for the remainder of the study for participant convenience. Interview data collected from participants was deidentified before data storage and analysis. The AFib Connect app was downloaded by research staff onto each participant’s personal mobile phone for use throughout the study period. Any protected health information that a participant may have entered into the app was securely stored locally on their phone and was not accessible to the researchers. Table 2 provides an overview of the study design and data collection schedule.

### Semistructured Interviews

A total of five interviews were conducted with each participant at weekly intervals over the 4-week study period. During enrollment, participants were asked background questions regarding their AFib history, overall technology use, and expectations about using an app for AFib. Participants were then asked to explore the app on their mobile phones and provide their initial impressions of each feature and the app overall. Observations about the participant’s interaction with the app were noted by the researchers.

From week 1 to 3, participants were asked to explore 2 or 3 predetermined features in detail in addition to the app overall. A list of these features and brief instructions were emailed to participants 1 week before the interview as a reminder. Phone interviews lasted 30 min, and participants were asked to discuss and rate the features they tested over the past week and the app overall in terms of its usability and current usefulness to them. During week 4, participants were asked to again review the entire app and provide feedback and a rating on their overall experience. Throughout the interviews, participants were encouraged to provide their honest and candid feedback about the app. Researchers paid close attention to conversational tone and pauses and asked follow-up questions, where needed, to probe more deeply into participant’s responses and to minimize any respondent bias.

Each interview was attended by two qualitative researchers, with one researcher leading the interview and the other taking detailed notes. Interviews were audiorecorded, and transcriptions of each recording were generated. At the completion of every interview, notes were discussed and summarized by the researchers. Utilizing grounded theory, a coding framework was developed from the interview questions. At regular intervals throughout the study, emergent codes were derived from note summaries and interview transcripts. After study completion, codes from each interview were compared and then organized into themes to derive the final results.

### Surveys

Supplementary study data was collected by custom surveys previously developed by Partners Connected Health. At study enrollment, information on participant demographics and technology use was collected. At closeout, patient satisfaction and app usability was measured with a 5-point Likert scale, yes or no, and open-ended questions.

Survey data was analyzed for the 12 participants who completed the study. Demographic, technology use, patient satisfaction, and usability characteristics were summarized. Descriptive statistics were reported as means and standard deviations for continuous variables and as percentages for categorical variables.

**Table 2:** Study design and data collection schedule.

Study visit and interviews		Surveys
**Enrollment (in person)**		
	Medical and AFib^a^ history	Demographics
	Current use of technology	Technology use
	Expectations from app	
	Initial impression of each feature and app overall	
**Week 1^b^ (by phone)**		N/A^c^
	AFib Guide	
	Medication Reminder	
	Additional feedback by feature and app overall	
**Week 2^b^ (by phone)**		N/A
	Heart Rate Monitor	
	Episode Tracker	
	Library	
	Additional feedback by feature and app overall	
**Week 3^b^ (by phone)**		N/A
	Trigger Tracker	
	News Feed	
	Appointment Reminder	
	App overall	
**Closeout (by phone)**		
	Needs when first diagnosed	Satisfaction and usability
	Likelihood to use after study	
	Likelihood to recommend to others with AFib	
	Additional feedback by feature and app overall	

^a^AFib: atrial fibrillation.

^b^Detailed feedback on the features scheuduled for data collection.

^c^N/A: not applicable.

## Results

### Participant Characteristics

The study comprised 7 males and 5 females, ranging in age from 37 to 67 years, with a mean of 59 years. Participants had been managing their AFib for 6 years on average, with a range from 1 to 15 years, and none were newly diagnosed. Eleven out of 12 participants (92%) were asymptomatic at the time of study participation because of having an ablation or cardioversion procedure, or a diagnosis of persistent AFib. One participant (8%, 1/12) experienced an AFib episode during the study. Table 3 provides a summary of characteristics for the study participants.

### App Usability

Results from survey data showed that all 12 (100%, 12/12) participants somewhat or strongly agreed that the app was easy to use and navigate, with 9 (75%, 9/12) stating they always knew what to do in the AFib app, and only one (8%, 1/12) reported needing to ask for help while using the app. Ten participants (83%, 10/12) somewhat or strongly agreed that the AFib app acted and felt like other apps they had used before.

Interview data revealed a positive perception of app usability; however, participants identified a few areas that could be improved to provide a better overall user experience. Areas of usability improvement can be organized into 3 categories: navigation, clarity of instructions and design intent, and software bugs.

#### Navigation

Participants stated that finding key features and the navigation between screens of the app was simple and straightforward:

I thought it was well designed as an app in that it sort of follows the typical style of most apps, so you don’t really—it’s easy just to touch things and you understand quickly what you need to be doing...I thought it was very well designed in terms of navigation.

Participant 5

However, one participant (8%, 1/12) reported difficulty discovering some of the AFib video content and another could not locate the news feed. There was also a reported disruption of workflow when, after reviewing an article in the Library and returning to the Library home screen, users were brought to the top of the page rather than to the section where they had left off.

#### Clarity of Instruction and Design Intent

Additional instruction, or clearer design intent in some areas of the app, might also improve the app’s overall usability. During app set-up, the researchers observed that nearly all the participants questioned whether certain data fields were required or optional and the type of information they should to enter in the medication notes field.

Taking a heart rate reading using the mobile phone camera was a novel and liked concept for nearly all the participants; however, many individuals expressed uncertainty about how the feature worked, where to place their finger on the camera flash, and whether they were taking their heart rate correctly. Nine of the 12 (75%) participants mentioned that having step-by-step illustrations of how to use the feature and additional context on how to interpret and act on readings would be helpful:

...It would be helpful if, within the app, there was some information like [normal heart rate range and heart rate range after an ablation] because as I’m taking my heart rate, I’m thinking, “My resting heart rate is supposed to be around 60. Now it’s 80.” So, I had to go outside of the app to get that information.

Participant 7

The Trigger Tracker was another feature that participants were initially uncertain how to use. Although the feature was designed to allow users to log their triggers upon exposure to establish a trigger history, 7 of the 12 (58%) participants assumed that they would note potential triggers retrospectively only after an episode occurred:

I guess I was a bit confused as to the purpose of this. I read the information a couple of times and I sort of walked away unclear. I mean, I could make assumptions, but I sort of walked away unclear. It seemed like you’re asking me to input triggers so that I can determine what my triggers are.

Participant 15

Potentially, because of assumptions about intended use or similarities in naming, there was also some confusion about the difference between the Trigger Tracker and the Episode Tracker, with 2 participants (25%, 2/12) referring to them as if they were the same feature.

**Table 3:** Summary of characteristics for the 12 study participants.

Participant characteristics		Value
Age in years, mean (SD)		59.25 (7.78)
Time since AFib^a^ diagnosis (years), mean (SD)		5.67 (4.54)
**Gender, n (%)**		
	Male	7 (58)
**Race, n (%)**		
	Caucasian	12 (100)
**Education, n (%)**		
	12 years or completed high school or general educational development	1 (8)
	Some college	2 (17)
	College graduate	3 (25)
	Graduate or professional degree	6 (50)
**Employment, n (%)**		
	Employed	4 (33)
	Homemaker	1 (8)
	Self-employed, full or part-time	3 (25)
	Retired	4 (33)
**Mobile phone type, n (%)**		
	Android	3 (25)
	iPhone	9 (75)

^a^AFib: atrial fibrillation.

#### Software Bugs

Seven out of 12 (58%) of participants reported software bugs that negatively affected their experience with the app. Participants on Android devices reported occasional inconsistent readings or app crashes when using the Heart Rate Monitor. Participants who reported this issue assumed the readings were inaccurate, with 1 participant (8%, 1/12) indicating that seeing this fluctuation caused some minor anxiety. The app error was immediately corrected to prevent further issues, and all the participants were given additional verbal instructions on how to take heart rate readings correctly, as unclear app instructions may have partly contributed to the variation in values.

Two participants (25%, 2/12) also reported a bug on the Appointment Reminder and Medication Reminder calendars, which caused the date drop-down fields to be cut off from view. Although the feature was still usable despite the bug, the affected participants reported a less user-friendly experience because of the issue.

### App Usefulness

Overall, 92% (11/12) of participants reported being satisfied or very satisfied with the AFib Connect app, and all the participants indicated that they were somewhat or very likely to recommend the app to people in treatment for AFib. Ten out of 12 (83%) participants somewhat or strongly agreed that they found the AFib app useful as a tool to track AFib related information, manage medical appointments, and be reminded to take their medication:

I’ve had AFib for a year and a lot of the information that I received, [was through my own] efforts of research, and a lot of the tracking information that I needed to monitor my condition was done by hand. [This app] is a convenient way and tool to keep things organized, to handle appointments and reminders, and to interface between yourself, your condition, and your caregivers.

Participant 3

[By study closeout] I continued to really just utilize the reminder feature, which works well. [It] works just the way I need it to work for me, for my personality, which is it bugs me until I don’t want it to bug me anymore so I take my pills. And frankly, for me, the app’s worth having just for that.

Participant 5

Although participant perceptions of the app’s usefulness for supporting self-care and medication adherence were largely positive, several areas of improvement were identified. The following outlines participant perceptions of usefulness for each of the app’s eight features and the app overall.

#### Library

The Library was rated highly in terms of usefulness to participants. Participants reported that the content was clear, relevant, comprehensive:

I think that [there is] a lot of good basic information...it’s easy to understand and informative enough for anyone who has AFib problems. It pretty much covers everything about it.

Participant 14

Several participants (58%, 7/12) reported that while they were familiar with most of the Library content and might not use it every day, it was still a nice resource for them to reference and refresh their knowledge. Five participants (42%, 5/12) also requested more treatment-related information, including comparative data between newer AFib medications and procedures.

#### Medication Reminder

Participants also responded positively to the Medication Reminder feature. Ten participants (83%, 10/12) reported that the AFib app helped them keep track of taking their medications. Seven (58%, 7/12) reported using the feature about once a day. More than half of participants reported that even without the AFib app they would have remembered to take their medications every day because of having an existing means or habit of remembering to take their medication:

But I think I just like having a reminder that pops up every day when I’m supposed to take the meds, I think that’s a neat feature...Even though I’d always remember, it’s just nice to have it pop up and remind you.

Participant 10

One participant (8%, 1/12) identified an additional use for the Medication Reminder feature, indicating that she would use it to keep track of all her medications and dosage information, beyond those she takes for AFib, so that she has this information readily accessible when needed.

#### Heart Rate Monitor

Despite the usability issues noted previously, participants liked the ability to check their heart rate quickly and easily and keep a history of their readings on their mobile phone. Although 11 out of 12 participants (92%) did not experience episodes during the study period, all of them reported checking their heart rate periodically throughout the day, or after exercise:

...[AFib patients] want to make sure that their heart rate is nice and even and down where it should be. When it’s out of whack, that’s a good indicator that you’re going into AFib. Some people don’t really know they’re in AFib unless they check that. So I think that’s an important part of your application.

Participant 9

#### AFib Guide

AFib Guide information about the different types of medication and procedures for AFib would have been especially useful to patients when they were first diagnosed. Participants reported receiving some education about AFib from their doctor; however, most of them indicated that they had to do a lot of their own Internet research, which took some effort. As, on average, our study sample had been living with AFib for 6 years, the Guide was used as more of a reference to review information:

It has a lot of good information in there. A lot of the information I did know already, but some other things that I did not know. I thought it was kind of interesting that you could make a list of what was important to you, and to go over with the doctor...It made me more aware of the details of AFib, what happens to you. But all of the treatments and the reasons why you have ablation, I was already very familiar with...For me, I would still want to keep it because I just think it’s a good review.

Participant 14

Participants reported that the AFib Guide’s videos and animations were especially helpful in explaining the content. Additionally, participants liked the idea of emailing their AFib Guide results to a doctor, although they indicated that they would more likely reference this record at an in-person visit.

#### Episode Tracker

As our study participants were asymptomatic at the time of the study, the Episode Tracker feature was not relevant to their current condition. Still, participants liked the idea of tracking an episode’s duration and making notes of what happened and felt it would have been a useful record-keeping tool when they were first diagnosed:

I would definitely use the episode tracker because that would eliminate my need for writing lengthy notes on my iPhone. So, I thought that the episode tracker was set up very well, and would probably shorten the length of time needed to get the information down where I could access it quickly and possibly reference it or email it to the appropriate party...I’m not using it now because I’m not having episodes. When I was having episodes, I believe I would use it.

Participant 3

Most participants indicated that if they believed they were having an episode they would first take their heart rate to verify if it’s elevated; if yes, they would then begin recording the episode’s duration and make note of any potential triggers. One participant (8%, 1/12) also suggested that the Episode Tracker include information to help individuals get through an episode, for example, by encouraging them to breathe slowly.

#### Trigger Tracker

Although participants liked the idea of the Trigger Tracker, this feature had less relevance to them during our study as most were not experiencing episodes. Some individuals also suggested that the Trigger Tracker feature would be more useful if it was combined with the Episode Tracker features, so that a history of their information is in a single place:

The way the Trigger Tracker is set-up I don’t find that helpful at all. I think unless it interfaces with the episode tracker, for me, I don’t see how it's helpful...There’s no relevance to an episode.

Participant 7

#### Appointment Reminder

Participants liked the idea of an Appointment Reminder tool where they can keep track of AFib and other medical appointments. However, most participants already used other tools to keep track of their schedule and seemed unlikely to adopt the AFib Connect Appointment Reminder unless there is an easy way to sync appointment information between their personal calendar or electronic medical record.

#### News Feed

Participants tended to rate the News Feed feature lower than the app’s other features. Although some participants liked the idea of having access to the latest heart health-related research and information, many felt the News feed content was not tailored enough to their specific needs.

#### App Overall

Participants’ perceptions of the app’s overall usefulness can be organized into three key themes: the needs of the patient segment, how well the app’s design supports the patient workflow, and whether the app can support the patient over time.

Although the target population of this study was any nonvalvular AFib patient who has been prescribed an NOAC, our data identified a few distinct patient segments within this group: (1) newly diagnosed patients versus those who have been managing AFib for an extended period and (2) patients who are otherwise healthy versus those with multiple comorbidities. As all the participants in our study had been managing AFib for more than a year, they expressed that, overall, the AFib Connect mobile app would have been significantly more useful when they were first diagnosed, still learning about AFib, and still experiencing symptoms:

Again, it’s more for the medication, appointment reminders, maybe trigger tracker kind of things because most of the other stuff I’ve been through and so I have a pretty good knowledge of the condition now. So I don’t think I’d be going back to the library much. I’m not going to obviously go back to the AFib guide, nor the heart rate monitor.

Participant 10

Similarly, although our study participants were relatively young and had few health conditions apart from AFib, previous research has shown that on average AFib patients are older and have multiple comorbidities [1,19]. We assume that older individuals and those managing multiple conditions would identify different needs for the app than the sample in our study.

Participant feedback also indicated that the app’s overall usefulness is impacted by how well the designed path through the app matches their natural workflow. For example, some participants suggested having the Heart Rate Monitor, Trigger Tracker, and Episode Tracker data and features interface more seamlessly to more easily track key data. One participant also suggested syncing the app’s data to their online medical record so that all their AFib health information can be accessed from a single place:

I don’t know if there’s ever going to be a way to get it so that it interfaces or interacts with the [online medical record] so that maybe you come in and you log in and then somehow it ties into all your information that’s there.

Participant 10

Study data also highlighted how patient perceptions of the app may shift over time. Participants discussed in detail how their needs now differ greatly from when they were first diagnosed and still experiencing symptoms. Similarly, we expect that as an AFib patient gets older, or experiences an improvement or deterioration in their condition, that their health priorities and needs will also change [20]. Whether the app can continue supporting a patient’s evolving needs will greatly impact its overall usefulness.

## Discussion

### Principal Findings

Results from this study provided greater insight into patient use and acceptability of the AFib Connect mobile app. Additionally, it helped paint a more complete picture of the everyday experience of AFib patients. By giving participants the opportunity to use the app in their natural environment and using qualitative research methods to explore perceptions of usability and usefulness, we obtained valuable feedback on its key features, navigation, content, and workflow that can be used to improve the overall design.

Additionally, study results illustrate three design principles that can be applied more broadly across the development of patient health apps: understand exactly who you are designing for, understand the patient’s natural workflow, and understand how patient needs change over time.

#### Understand Exactly Who You Are Designing For

This study demonstrates how the needs of patients can vary depending on how long they have been managing their condition and whether they have additional comorbidities. Understanding what patient segments exist within the larger population of individuals who share a medical condition and designing for their unique needs is essential to building useful and usable apps. Feedback from all participants indicated that the app would have helped them manage their AFib care and treatment in general and significantly more so when they were first diagnosed.

#### Understand Patient Workflow

Qualitative feedback from participants also revealed exactly how features will be used, when they will be used, and how this app will fit within the broader ecosystem of tools and information resources patients access to manage AFib. It became clear after several interviews that participants might have a more streamlined experience if some app features were combined to better reflect the natural workflow of AFib patients. By considering not only the usefulness of each individual feature, but how these features work together to support self-care and treatment adherence, is key.

#### Understand How Needs Change Over Time

Results from this study also indicate that the usefulness of a health app often changes over time, largely based on a patient’s changing health status and knowledge of their condition. Similarly, we suspect a user’s interaction with a health app may evolve the longer they have used the app and are familiar with its content. For a health app to continue to be useful over a long period, its design will need to consider and adapt to the changing needs of its users.

To build more useful and usable tools for self-care and treatment adherence, it is essential to holistically examine the context in which patients experience their condition. We should evaluate whether the app truly meets the core needs of the target population, if it fits into their natural workflow and with the tools they already use, and whether it can continue to provide support throughout each stage of their condition.

### Limitations

This exploratory pilot study has a few limitations. A key limitation was the study sample. Participants were younger than the typical AFib population [1], all Caucasian, and none were newly diagnosed. Additionally, as most of the participants did not experience episodes during the study, much of the usefulness feedback we received was based on how the app’s features would have been useful in the past, and thus, responses may be influenced by recall bias. Although the results of exploratory studies are not intended to be generalizable, additional future research utilizing a larger and more diverse sample of newly diagnosed AFib patients will be helpful for understanding the app’s wider applicability.

Another limitation was the duration of the study. Although this study was useful for gaining insight into the AFib patient experience and perceptions of the app over a 4-week period, it would be valuable to see if these perceptions change over a longer period of naturalistic use, as patient health conditions and needs evolve.

### Comparison With Prior Research

Mobile tools to support self-care and medication adherence have previously shown promise in supporting the patient management of chronic conditions [20]; however, this study is the first we are aware of that specifically examines how these tools might be useful for AFib.

The benefits of qualitative methods for gaining rich insight into the real-world use and acceptability of health apps are well documented [21,22]. Additionally, the value of incorporating patient perspectives during the early stages of design and testing of a new intervention is supported by a growing body of research [23,24]. Yet, relatively few medical studies use qualitative research methods to examine patient perceptions of an intervention at an early stage, or at all, before implementation [23]. This can result in less than optimal, or even negative outcomes for patients who receive the intervention [24].

### Conclusions

The results of this study suggest that mobile tools that target self-care and treatment adherence may be helpful to AFib patients, particularly those who are newly diagnosed. Additionally, participant feedback provided insight into the varied needs and health experiences of AFib patients, which may improve the design and targeting of the intervention.

Pilot studies that qualitatively examine patient perceptions of usability and usefulness are a valuable and often underutilized method for assessing the real-world acceptability of an intervention [25,26]. Additional research evaluating the app over a longer period and including a larger, more diverse sample of AFib patients will be helpful for understanding whether the AFib Connect mobile app and similar tools can be more widely useful.

By expanding our understanding of the AFib patient experience, we can continue to improve the app’s usability and usefulness and its capability for supporting long-term self-care and treatment adherence.

## Appendix

Multimedia Appendix 1 A sample of questions from the Week 2 Semistructured Interview Guide.

## Abbreviations

AFib: atrial fibrillation
NOAC: nonvitamin K antagonist oral anticoagulants
