# Supplementary material for: Evaluating the Usability and Usefulness of a Mobile App for Atrial Fibrillation Using Qualitative Methods: Exploratory Pilot Study
Source: JMIR Hum Factors. 2018 Mar 15;5(1):e13. doi: 10.2196/humanfactors.8004 (PMC5876493; doi:10.2196/humanfactors.8004)
Supplement: Multimedia Appendix 1 [file humanfactors_v5i1e13_app1.pdf]

AFib Connect

Semi-Structured Interview Guide

(Note: The questions below are a sample of the types of information that was discussed during Week 2 of the semi-structured interviews. These serve only as a guide to the discussion, and are not comprehensive of all the questions that may have been asked in the course of the conversation.)

Week 2

Introduction

Thanks for taking the time to speak with us this week.

Last week we asked you to:

Explore the **Heart Rate Monitor, Episode Tracker** and **Library**

For this week's discussion we'd like to get your feedback on the app overall, and the features you tried this week in particular. As a reminder, we want your honest and candid feedback about the application. Please feel free to share what you like, don't like, and any suggestions for improvement.

General reflection:

- What features did you use?
    - Why did you use them?
    - When did you use them?
    - How frequently?
- What did you like?
- What didn't you like?
- Were any of these features useful to you?
- Any difficulties using these features?
- Any suggested changes?

Heart Rate Monitor:

Now I'd like to ask about your experience with the **Heart Rate Monitor**

- How do you feel overall about the Heart Rate Monitor?
- What did you like?
- What didn't you like?
- Was this feature useful to you?
- How did you use this feature?
- Any difficulties using the feature?
- Any suggested changes?

Episode Tracker:

Let's talk about the **Episode Tracker**.

- How do you feel overall about the Episode Tracker?
- Were you able to enter a new episode?
- Were you able to end an episode?
- Were you able view this episode in the History tab?
- What did you like?
- What didn't you like?
- Was this feature useful to you?
- How did you use this feature?
- Any difficulties using the feature?
- Any suggested changes?

Library:

The last feature we asked you to try this week was the **Library.**

- How do you feel overall about the Library?
- Was the information within the library valuable to you?
  - Why or why not?
- What did you like?
- What didn't you like?
- Was this feature useful to you?
- How did you use this feature?
- Any difficulties using the feature?
- Any suggested changes?

Next Steps

Thank you for your feedback!
Over the next week, we would like you to explore the **Trigger Tracker** and the **Appointment Reminder.**
Could you look through the app right now and see if you can find these two features?

Interviewer will guide participant to above features if needed

Additional information about these features will also be sent to you by email in the next 24 hrs.
Please feel free to reach out to us with any questions if needed.
We will contact you in one week to follow-up on these tasks. The conversation should take about 30 minutes.
